# An HIV‐positive woman with massive brain lesion due to toxoplasmosis: A case report

**DOI:** 10.1002/ccr3.7688

**Published:** 2023-07-13

**Authors:** Reza Ghasemikhah, Zahra Hakimzadeh, Abolfazl Gilani, Hossein Sarmadian, Roham Sarmadian, Negin Yousefbeigi

**Affiliations:** ^1^ Department of Parasitology & Mycology Arak University of Medical Sciences Arak Iran; ^2^ Neurosciences Research Center (NSRC) Tabriz University of Medical Sciences East Azerbaijan Iran; ^3^ Sina Trauma & Surgery Research Center Tehran University of Medical Sciences Tehran Iran; ^4^ Department of Infectious Diseases Arak University of Medical Sciences Arak Iran; ^5^ Infectious Diseases Research Center Arak University of Medical Sciences Arak Iran; ^6^ Department of Radiology Arak University of Medical Sciences Arak Iran

**Keywords:** brain lesion, case report, HIV, opportunistic infection, toxoplasmosis

## Abstract

**Key Clinical Message:**

Toxoplasmosis‐related huge brain lesions may require decompressive craniectomy and lesion excision to avoid brain damage. In this situation, injectable cotrimoxazole is a better choice for treatment.

**Abstract:**

Toxoplasma gondii is a worldly distributed obligate intracellular protozoa. Toxoplasmosis is a prevalent opportunistic infection in HIV‐infected people, but it was rarely recorded prior to the identification of HIV infection. Here, we report a toxoplasmosis brain lesion in an Iranian HIV‐positive patient. A 45‐year‐old woman with a complaint of malaise was referred to the Valiasr Hospital in Arak city. In her past clinical history, the patient had a history of anemia, deep vein thrombosis (DVT), and positive HIV. The patient was informed of the diagnosis of massive brain toxoplasmosis as a definite diagnosis. The patient was then taken to the operating room for a left decompressive craniectomy, during which the ensuing brain lesion was excised. After a few days, she was discharged from the hospital in good condition and without any complications.

## 1 INTRODUCTION

Toxoplasmosis is an obligate intracellular, nutrient‐borne parasitic disease. The parasite is transmitted through the consumption of raw or uncooked meat contaminated by feline feces, the definitive host of oocytes.^1^ Toxoplasma gondii (T. gondii) is commonly propagated asexually in humans by consuming tissue cysts containing bradyzoites; it can, however, be transmitted through the consumption of oocysts containing sporozoites found in the intestines and feces of cats during their sexual cycles. Consumption of undercooked meat is the classical major risk factor for toxoplasmosis infections.^2^ Almost 60% of the adult population is seropositive for this parasite that is capable of infecting all sorts of mammals.^3^

Most T. gondii infections are asymptomatic; people developing parasitic toxoplasmosis show no signs of infection if they have an intact immune system.^4^ However, clinical manifestations of the disease called toxoplasmosis can be seen in some individuals.^5^ Toxoplasmosis may involve lymph nodes, eyes, or the central nervous system.^6^ Toxoplasmosis can be life‐threatening in immunocompromised patients.^7^

Cerebral toxoplasmosis is an opportunistic infection seen in HIV‐infected patients. Prior to the emergence of HIV, this infection was extremely rare.^8^ Here, we report a toxoplasmosis brain lesion in an Iranian HIV‐positive patient.

## 2 CASE PRESENTATION

A 45‐year‐old woman with a complaint of malaise was referred to the Valiasr Hospital in Arak city. Upon arrival, the vital signs were heart rate of 78 per min, blood pressure of 125/80 mmHg, respiratory rate of 20 breaths per minute, oxygen saturation of 95% in ambient air, and axillary temperature of 36.8°C. Her level of consciousness was assessed according to the Glasgow coma scale (GCS) with a score of 9 out of 15 (Verbal 2, Motor 5, and Eye 2). The size of the pupils was normal, and they reacted to light equally.

The patient had a history of anemia, DVT, and positive HIV. She did not mention any high‐risk behaviors and did not have a pet such as a dog or a cat.

**FIGURE 1:**
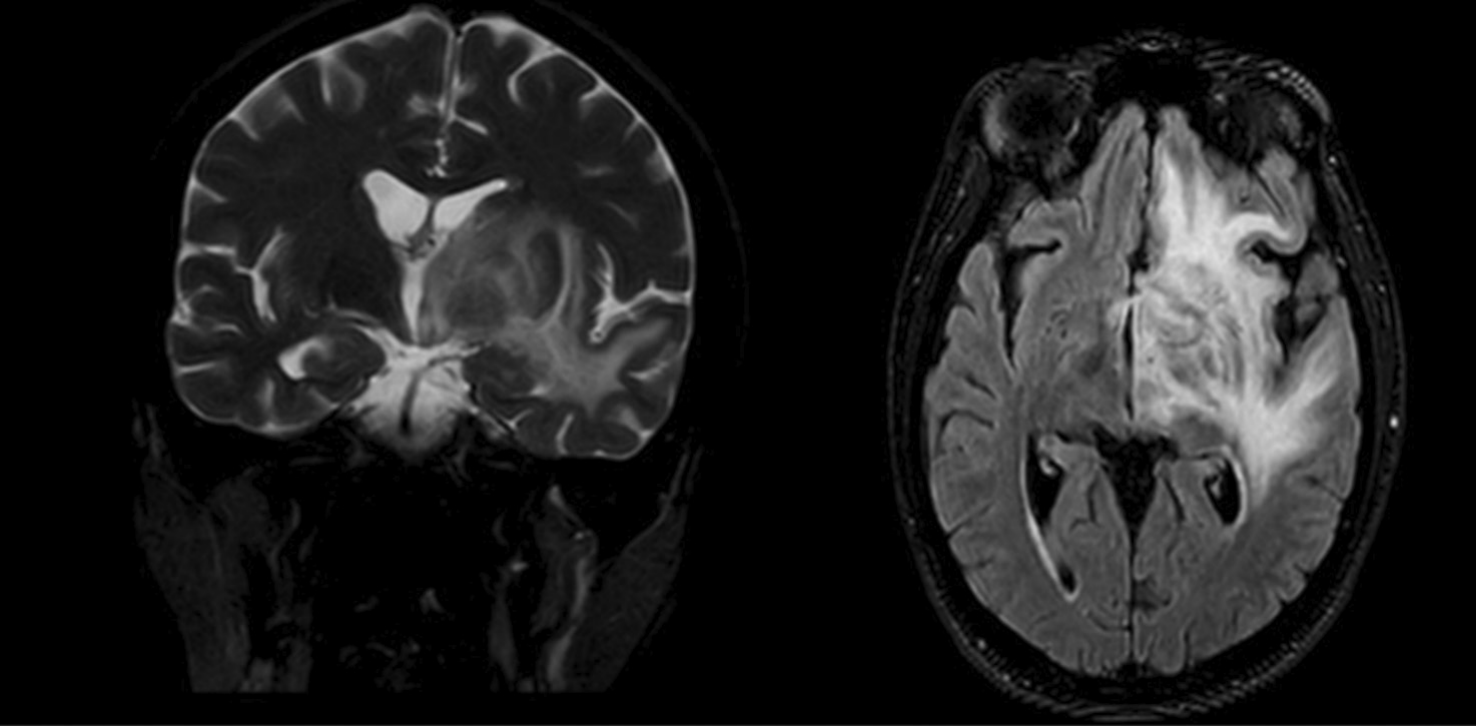
Brain lesion due to toxoplasmosis (left frontoparietal region).

The patient was undergone clinical and paraclinical assessments, and different diagnoses were made. The brain MRI reported a brain mass in the left frontoparietal region (Figure 1).

The physician ordered a PCR examination of the patient's cerebrospinal fluid (CSF) based on this report. A sample was taken from the patient and sent to the laboratory to test for aspergillus, candidiasis, EBV, CMV, staphylococcus, T. gondii, and HSV using PCR. Among the listed microorganisms, the presence of T. gondii was confirmed. According to the type of brain lesion seen in the left frontoparietal region of the brain, the diagnosis of toxoplasmosis was made conclusively.

The patient was then taken to the operating room for left decompressive craniectomy, which involved the removal of the brain lesion. Because of the increased intracranial pressure and to avoid brain tissue injury, an abdominal fat graft was performed, and the skull was put within the abdomen fat. The guideline for the treatment of AIDS patients with cerebral toxoplasmosis is pyrimethamine + sulfadiazine + folic acid, but according to our patient's condition after brain surgery, the patient was treated with an injectable form of cotrimoxazole.

After a few days, when cranioplasty was performed on the patient and the patient was generally healed, she was discharged from the hospital with a good general condition and without fever or other complications.

## 3 DISCUSSION

The most common CNS infection in HIV‐infected patients is cerebral toxoplasmosis, which should always be suspected in immunocompromised patients who present with new neurological symptoms.^9^, ^10^

Toxoplasmosis may be diagnosed clinically, radiologically, by serologic tests, histologically or by molecular methods. Detection of T. gondii in CSF or peripheral blood samples of patients with expansive brain lesions who also have other opportunistic neurological diseases can help with the timely diagnosis. Toxoplasmosis is most commonly manifested in HIV‐positive patients as toxoplasmic encephalitis, which is one of the causes of localized CNS lesions. Toxoplasmic encephalitis is associated with headaches, lethargy, altered behavior, and fever. Speech disturbance and motor weakness are the two most prevalent focal neurological signs. Seizures, cranial nerve disturbances, visual field defects, sensory disorders, cerebellar dysfunction, meningismus, movement disorders, and neuropsychiatric manifestations have all been reported in these patients.^11^, ^12^ Our case presented with altered mental status and generalized malaise as reported in other studies. Patients with cerebral toxoplasmosis frequently present with pulmonary and ocular disorders. Rarely, toxoplasmosis can progress to a rapidly fatal type of diffuse encephalitis.

Although anti‐T. gondii IgM antibodies are frequently used in serologic tests, the gold standard is the detection of IgG antibodies with the Sabin–Feldman dye test. Patients with toxoplasmic encephalitis may have elevated protein levels and mild pleocytic mononuclear predominance in their CSF.^12^, ^13^ Detection of T. gondii DNA using polymerase chain reaction (PCR) has a sensitivity of 12–70% and specificity of 100% in toxoplasmic encephalitis patients.^14^ According to the report in our study, T. gondii was identified among the potential microorganisms in the patient's CSF PCR test. T. gondii isolation from blood or body fluids such as CSF and bronchoalveolar lavage fluids, as well as tissue biopsies, can also be used to diagnose toxoplasmosis. Although CT and MRI cranial imaging have no pathognomonic value, the distribution or appearance of brain lesions may provide helpful information. In between 70 and 80 percent of patients, CT scan can detect multiple, bilateral, hypodense contrast‐enhancing focal brain lesions. Typically, basal ganglia and the hemispheric corticomedullary junction are affected by brain lesions in toxoplasmic encephalitis.

A ring‐like pattern can be observed around the lesion using contrast enhancement. On a CT scan, toxoplasmic encephalitis may occasionally show a single lesion or no lesions at all,^15^ whereas MRI can be used as a more sensitive imaging method, especially in patients with focal neurological abnormalities. MRI is known as the best screening method for CNS toxoplasmosis, however, when a diagnosis is unclear, a brain biopsy is a safe procedure that gives these patients high diagnostic yields.^10^ MRI scans of these patients frequently reveal multiple, bilateral, and ring‐enhancing lesions over the corticomedullary junctions of the cerebral hemisphere and basal ganglia. Solitary lesions can also be seen in 14% of cases. Multiple rings on an MRI, a positive IgG serology test for Toxoplasma, and a low absolute CD4 count of less than 200 cells/mm^3^ are indications for starting the empirical treatment for T. gondii.^16^ Repeating the imaging is recommended if the empirical treatment has already started without biopsy. MRI or CT scans can both be used for posttreatment follow‐up, but a biopsy is more characteristic if they remain unchanged. In our case, the distribution of the lesions and the emergence of “target lesions” were both typical of toxoplasmic encephalitis.

Dynamic MRI scans are crucial for monitoring outcomes during treatment courses. Reduction in MRI abnormalities and positive clinical dynamics within 2 weeks following the therapy indicate therapeutic progress.^17^ We employed MRI imaging to track the response. After reporting a definitive diagnosis of toxoplasmosis due to the type of brain lesion seen in the left paralytic anterior region of the brain, the patient was transferred to the operating room to remove the brain lesion using left decompressive craniectomy.

Prophylaxis is advised for HIV patients with positive Toxoplasma IgG and a low CD4 cell count, which may be discontinued if the CD4 cell count returns to greater than 200 for at least 6 months. HIV patients should be informed about possible exposure to Toxoplasma and tested for Toxoplasma IgG antibodies to detect latent toxoplasmic infections. Toxoplasmosis can be prevented by avoiding raw or undercooked meat, personal hygiene, and avoiding animal waste. Recently multiple drug combinations were tested in clinical trials for the treatment of cerebral toxoplasmosis, but according to available evidence, the best regime has not yet been identified in terms of relative efficacy or safety. A review study found that pyrimethamine and sulfadiazine combination showed fewer relapses during maintenance therapy and developed as the most effective regime; however, another one concluded that the use of trimethoprim and sulfamethoxazole combinations has potential advantages and preferred treatment for cerebral toxoplasmosis in HIV‐infected adults as we saw a favorable result in the current case.^18^, ^19^ A case report also demonstrated that pyrimethamine, clindamycin, and steroids combination can increase the prognosis in cerebral toxoplasmosis cases with severe edema.^20^ Although further robust comparative studies are required in this area, the standard treatment regimen for toxoplasmosis encephalitis is a combination of sulfadiazine, folic acid, and pyrimethamine. A short course of corticosteroid treatment can also be used in toxoplasmosis along with the standard treatment. Due to our patient's postcranial surgery condition, she was treated with injectable cotrimoxazole (a combination of sulfamethoxazole and trimethoprim). Cotrimoxazole treatment was continued orally after discharge both for toxoplasmic encephalitis as well as for pneumocystis pneumonia prophylaxis. Generally, a methodical approach that includes quick diagnosis, suitable antimicrobial management, prompt ICU admission, and aggressive measures to decrease intracranial pressure may enhance the outcome.^9^

## 4 CONCLUSION

This report demonstrates that severe brain lesions, in some cases necessitating a craniectomy, can occur in HIV patients who did not receive timely toxoplasmosis prophylaxis or toxoplasmosis was not diagnosed and treated at an appropriate time. In addition, doctors are advised to regularly visit and examine patients. Performing neurological examinations in HIV patients is important. Moreover, regular CD4 count testing is also recommended. Furthermore, healthcare workers should ensure the use of prophylactic medications by patients.

## AUTHOR CONTRIBUTIONS

**Reza Ghasemikhah:** Conceptualization; investigation; project administration; resources; supervision. **Zahra Hakimzadeh:** Visualization; writing – original draft; writing – review and editing. **Abolfazl Gilani:** Investigation; supervision; writing – review and editing. **Hosein Sarmadian:** Supervision; validation; visualization. **Roham Sarmadian:** Project administration; supervision; visualization; writing – original draft; writing – review and editing. **Negin Yousefbeigi:** Methodology; validation; writing – original draft.

## FUNDING INFORMATION

This research did not receive any funding.

## CONFLICT OF INTEREST STATEMENT

The authors declare that they have no competing interests.

## ETHICS STATEMENT

Our institution does not require ethical approval for reporting individual cases or case series. All of the authors declare that the confidentiality of the patient was respected.

## CONSENT

Written informed consent was obtained from the patient for publication of this case report and any accompanying images.

## Data Availability

The data that support the findings of this study are available on request from the corresponding author.
